# Low-density lipoprotein mimics blood plasma-derived exosomes and microvesicles during isolation and detection

**DOI:** 10.1038/srep24316

**Published:** 2016-04-18

**Authors:** Barbara W Sódar, Ágnes Kittel, Krisztina Pálóczi, Krisztina V Vukman, Xabier Osteikoetxea, Katalin Szabó-Taylor, Andrea Németh, Beáta Sperlágh, Tamás Baranyai, Zoltán Giricz, Zoltán Wiener, Lilla Turiák, László Drahos, Éva Pállinger, Károly Vékey, Péter Ferdinandy, András Falus, Edit Irén Buzás

**Affiliations:** 1 Department of Genetics, Cell- and Immunobiology, Semmelweis University, Budapest, 1085, Hungary; 2 Institute of Experimental Medicine, Hungarian Academy of Sciences, Budapest, 1083, Hungary; 3 Department of Pharmacology and Pharmacotherapy, Semmelweis University, Budapest, 1085, Hungary; 4 Research Centre for Natural Sciences, Hungarian Academy of Sciences, Budapest, 1117, Hungary

## Abstract

Circulating extracellular vesicles have emerged as potential new biomarkers in a wide variety of diseases. Despite the increasing interest, their isolation and purification from body fluids remains challenging. Here we studied human pre-prandial and 4 hours postprandial platelet-free blood plasma samples as well as human platelet concentrates. Using flow cytometry, we found that the majority of circulating particles within the size range of extracellular vesicles lacked common vesicular markers. We identified most of these particles as lipoproteins (predominantly low-density lipoprotein, LDL) which mimicked the characteristics of extracellular vesicles and also co-purified with them. Based on biophysical properties of LDL this finding was highly unexpected. Current state-of-the-art extracellular vesicle isolation and purification methods did not result in lipoprotein-free vesicle preparations from blood plasma or from platelet concentrates. Furthermore, transmission electron microscopy showed an association of LDL with isolated vesicles upon *in vitro* mixing. This is the first study to show co-purification and *in vitro* association of LDL with extracellular vesicles and its interference with vesicle analysis. Our data point to the importance of careful study design and data interpretation in studies using blood-derived extracellular vesicles with special focus on potentially co-purified LDL.

Extracellular vesicles (EVs) are cell-derived submicron structures that have been gaining rapidly increasing attention in the past decade^1^,^2^,^3^. Although there is no consensus in the terminology, EVs that originate from multivesicular bodies and usually have ≤100 nm diameter are referred to as exosomes (EXOs)^4^, whereas plasma membrane-shed vesicles are either called microvesicles (MVs, 100–800 nm) or apoptotic bodies (>1 μm)^1^,^2^,^3^,^4^.

EVs have been found in various body fluids as well as in tissue culture supernatants^1^,^2^,^3^,^4^. Differences in concentration and composition of circulating EVs in human blood plasma have been shown to associate with various physiological and pathological conditions^1^,^5^,^6^,^7^,^8^,^9^. Circulating EVs originating from diseased tissues may serve as biomarkers^5^,^6^,^7^,^8^,^9^, and may also enter tissues and exert different functions^9^,^10^. The role of pre-analytical conditions has been demonstrated to influence data on circulating EVs^11^,^12^. However, the impact of food intake as a pre-analytical condition has not been addressed in detail yet.

Blood plasma contains a long known and extensively studied set of particles with a single phospholipid layer on their outside, which are known to show robust changes postprandially: lipoproteins^13^,^14^,^15^,^16^. Lipoproteins have been studied for decades and low-density lipoprotein (LDL) has been reported as a major risk factor in numerous cardiovascular diseases^15^,^17^,^18^.

Current enumeration techniques of circulating particles usually fail to discriminate between vesicular and non-vesicular structures such as protein aggregates^19^. In this study we set to analyze whether in addition to protein aggregates, lipoproteins also have an impact on the detection of EVs. High-density lipoprotein has already been identified as a possible contaminant of EV preparations^20^, and recently association of apolipoprotein E with melanocyte-derived exosomes has also been demonstrated^21^.

## Results

### Particle concentration within the size range of MVs increases significantly in blood plasma after a high-fat meal

**Figure 1:**
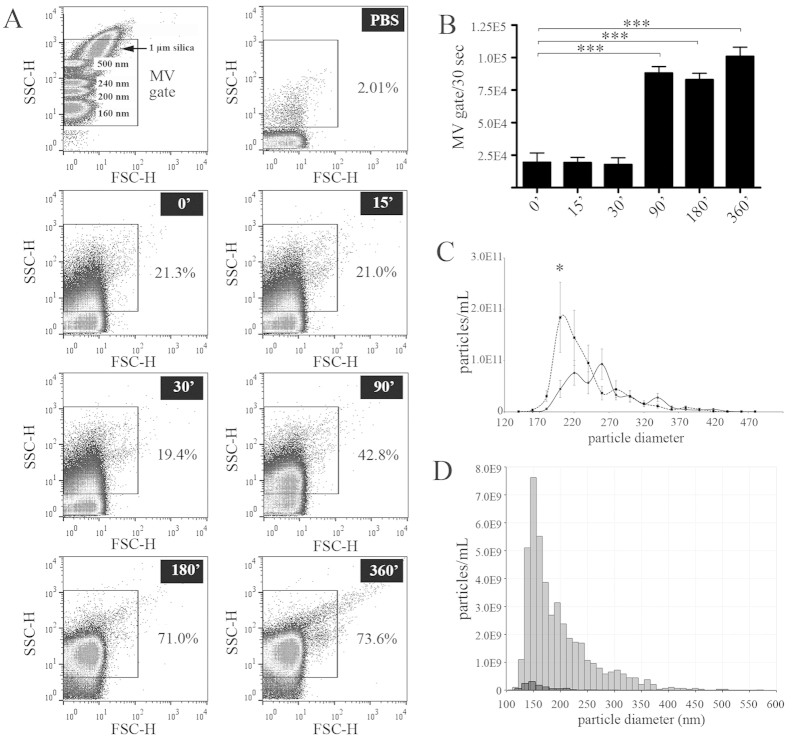
The impact of food intake on the number of blood plasma particles within the size range of EVs. (**A**,**B**) Representative scatter plots (**A**) and summarized data (**B**) of PFP samples from healthy donors (n = 3), analyzed by FCM in fasting state, 15 min, 30 min, 90 min, 180 min and 360 min after a standard high-fat meal. The MV gate was established using Megamix^TM^ beads (diameter: 160 nm–500 nm), and the gating was optimized with 1 μm silica beads. Note that the particle number within the MV gate significantly increased 90 min postprandially (mean + SEM, ***P < 0.001, one-way ANOVA), and remained elevated up to 6 h after food intake. (**C**) Representative data of TRPS analysis of fasting and 4 h postprandial human blood plasma samples (n = 3, continuous and dotted lines, respectively, *P < 0.05 Wilcoxon matched-pairs signed rank test). The samples were measured on NP200, NP400 and NP800 nanopore membranes. (**D**) TRPS measurement (NP200) of fasting (black bars) and 4 h postprandial (gray bars) plasma samples purified with SEC prior to TRPS analysis.

To study the effect that food intake has on the detectable particle concentration in blood plasma, platelet-free plasma (PFP) was collected from healthy individuals (n = 3) after 12 h fasting as well as at multiple time points postprandially, after a standard high-fat meal. Figure 1a,b show flow cytometry (FCM) scatter plots with a significant (up to 5×) increase in the detectable particle number within the MV gate. This increase became significant (^***^P < 0.001) after 90 min (Fig. 1b) and remained elevated even after 6 h. Therefore, in our subsequent experiments we chose to use 4 h postprandial PFP samples. As shown in Fig. 1c, TRPS analysis of fasting and 4 h postprandial PFPs revealed an increased postprandial particle concentration (*P < 0.05), however, without any significant alteration in the particle size (Supplementary Fig. S1). Similarly, tunable resistive pulse sensing (TRPS) analysis (a method suitable for measuring particle size and concentration) of PFPs purified on a qEV^TM^ size exclusion chromatography (SEC) column (Fig. 1d) maintained the prominent difference between fasting and postprandial samples, although there was a general reduction in particle concentration as compared to the unpurified samples (Fig. 1c,d).

### The majority of particles detected in PFP carry lipoprotein markers even in fasting conditions

**Figure 2:**
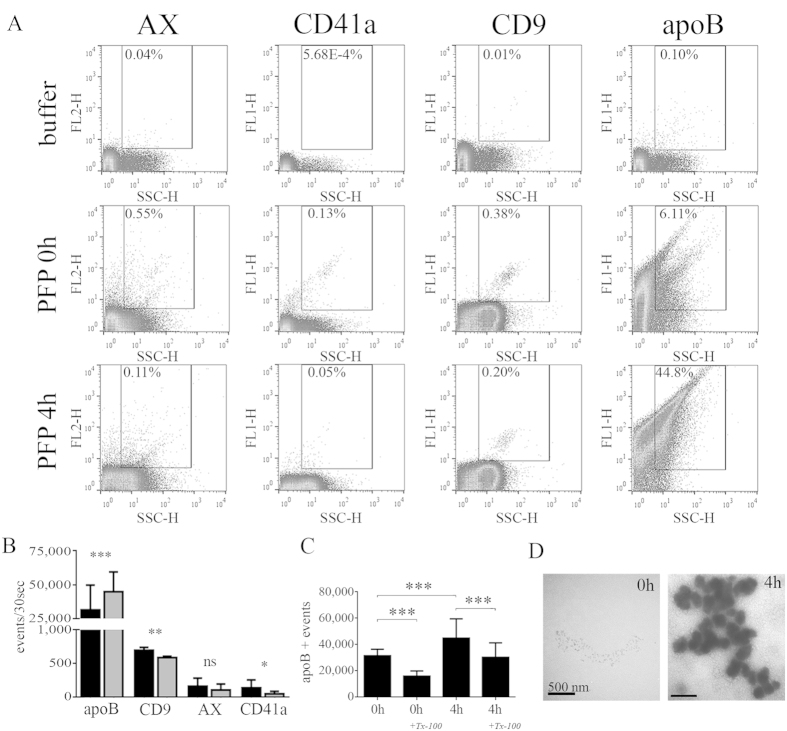
Analysis of particle nature in fasting and postprandial blood plasma samples. (**A**,**B**) Representative FCM plots (**A**) and their quantification (**B**) determined from 10 µL fasting (black bars) and 10 µL 4 h postprandial (gray bars) PFP samples of healthy individuals (n = 9, FCM, mean + SEM, ***P < 0.001, Wilcoxon matched-pairs signed rank test). CD9, AX and CD41a were used to identify MVs, and only events both staining for EV markers and sensitive to 0.1% Tx-100 were considered EVs. (**C**) Number of apoB-positive events within the MV gate in fasting (0 h) and postprandial (4 h) states, treated with Tx-100 (mean + SEM, ***P < 0.001, Wilcoxon matched-pairs signed rank test). (**D**) TEM analysis of the top 200 μL fractions of 2.5 mL ultracentrifuged (100,000 g, 2 h, 4 °C) fasting and postprandial plasma samples using an “osmification-on-grid” approach. Scale bar: 500 nm. Note that highly electron-dense floating particles were detected in the postprandial sample.

Next we addressed the question whether these particles that appeared in our FCM analysis postprandially, could be lipoproteins. Thus, we labelled fasting and 4 h postprandial PFPs (n = 9) with fluorescently labelled annexin V (AX) that binds phosphatidyl-serine, as well as anti-CD41a and anti-CD9 antibodies in order to detect EVs by FCM. Furthermore, we used an anti-apoB100/48 antibody to detect chylomicrons which have been reported to appear in blood ~4 h after food intake^16^,^22^ (Fig. 2a, fluorescence-based gating strategy: Supplementary Fig. S2). Surprisingly, only a few events within the MV size range carried EV markers, and even this low percentage of events was reduced postprandially (Fig. 2b, ^**^P < 0.01). In sharp contrast, most of the particles detected within the size range of MVs were recognized by an antibody against human apoB100/B48, even in fasting PFP samples. Importantly, the high number of apoB-positive events was significantly increased further in the postprandial state (Fig. 2b, ^***^P < 0.001). In order to identify EVs in our samples, we applied differential detergent lysis^19^,^23^. Only EV marker positive events which were disrupted in the presence of 0.1% Tx-100 were considered EVs, and are presented here. Importantly, we found that some of the apoB-positive events were also sensitive to detergent lysis even in fasting conditions (Fig. 2c, ^***^P < 0.001). However, the remaining apoB carrying events still highly outnumbered the disappeared EV marker-bearing ones (up to 20×). To confirm that lipoproteins appearing postprandially were chylomicrons, we tested whether they float upon ultracentrifugation (UC) as described previously^22^. To this end we analyzed the top fractions of ultracentrifuged fasting and postprandial PFPs by transmission electron microscopy (TEM). As shown in Fig. 2d, floating, electron-dense lipoproteins appeared 4 h after food intake. To correlate our findings with routine laboratory measurements, we measured serum triglyceride, total cholesterol, LDL cholesterol, apoA1 and apoB100 content. As shown in Supplementary Table S1, the concentration of triglycerides increased significantly (P < 0.05) in postprandial state. This further confirmed that these floating particles were chylomicrons which are known to be enriched postprandially in triglycerides^22^.

### MVs isolated from blood plasma and platelet concentrates carry substantial amounts of associated lipoproteins

**Figure 3:**
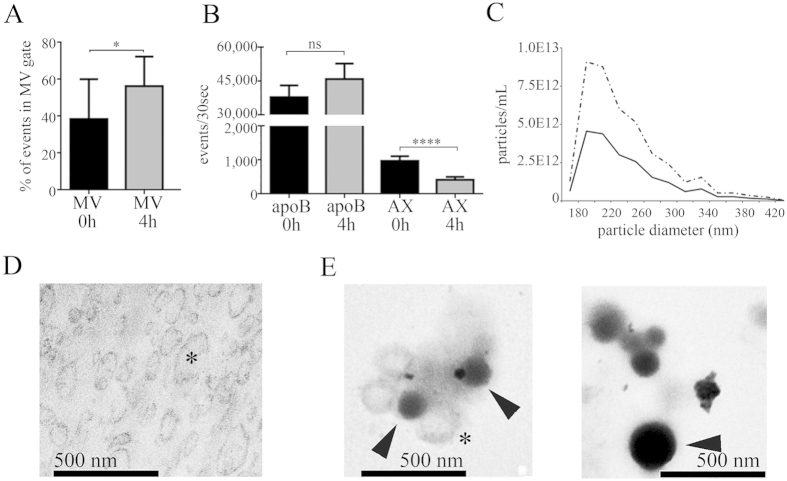
Detection of apoB-positive particles in isolated MV preparations. (**A**,**B**) MVs were isolated by differential centrifugation and gravity driven size filtration from 500 μL of fasting PFPs (black bars) and 4 h postprandial PFPs (gray bars) and analyzed by FCM. (**A**) The percentage of events detected within the MV gate increased significantly in the postprandial state (n = 9, mean + SEM, *P < 0.05, Wilcoxon matched-pairs signed rank test). (**b**) The isolated MVs were stained with anti-apoB and AX. For the AX labeling only 0.1% Tx-100 sensitive event s (which we considered EVs) were shown. The difference between the fasting and postprandial apoB-positive events was not significant, however, the number AX-positive events decreased significantly upon food intake (n = 9, mean + SEM, ****P < 0.0001, paired t-test). (**C**) Representative TRPS measurement of isolated fasting and postprandial MV preparations (continuous line: fasting MVs, dotted line: 4 h postprandial MVs). Note that the mean particle size was not affected. (**D**) TEM image of an ultrathin section prepared from a postprandial PFP-derived MV pellet (scale bar: 500 nm). Asterisk indicates MVs. (**E**) The same sample analyzed in suspension by an “osmification-on-grid” approach (scale bar: 500 nm). Note the highly electron-dense, round particles reminiscent to the TEM morphology of lipoproteins upon osmification (arrowheads). Membrane enclosed MVs (asterisk) showed association with these electron-dense particles.

Next we addressed the question whether lipoproteins carrying apoB were also present in isolated MV preparations. To test this, we isolated MVs from 500 μL PFP with differential centrifugation and gravity-driven size-filtration^11^,^19^,^24^. As shown in Fig. 3a, the percentage of events within the SSC-FSC based MV gate increased significantly in the postprandial state (P: 0.0195). However, there was no significant difference in the anti-apoB-staining of fasting and postprandial isolated MVs (Fig. 3b). This was most likely due to the fact that the already high amount of antibody (optimized for PFP staining) was still not sufficient to label all of the postprandially increased number of particles in MV preparations (Supplementary Fig S3). The AX-positive events decreased significantly upon food intake (P < 0.0001).

To confirm the vesicular nature of AX-positive events we performed detergent lysis^19^,^23^. Surprisingly, not only AX-, but also apoB-positive events showed partial sensitivity to 0.1% Tx-100 (Supplementary Fig. S4). We also used TRPS to assess the effect of food intake on the number of particles within the MV size range, and found a significant increase of particle concentration upon food intake (Fig. 3c and Supplementary Fig. S5).

Given that even in the fasting state the number of apoB-positive particles was at least a magnitude higher than the AX-positive ones, we further focused on the analysis and purification of fasting MV samples. We also analyzed isolated MVs by TEM, and found striking differences when using two different TEM approaches. While ultrathin sections of blood plasma-derived MV pellets (Fig. 3d) showed membrane enclosed vesicular structures, analysis of MV suspensions using the “osmification-on-grid” approach revealed highly electron-dense round structures within the size range of EVs and in close association with them (Fig. 3e) that were highly reminiscent to those shown in Fig. 2d.

**Figure 4:**
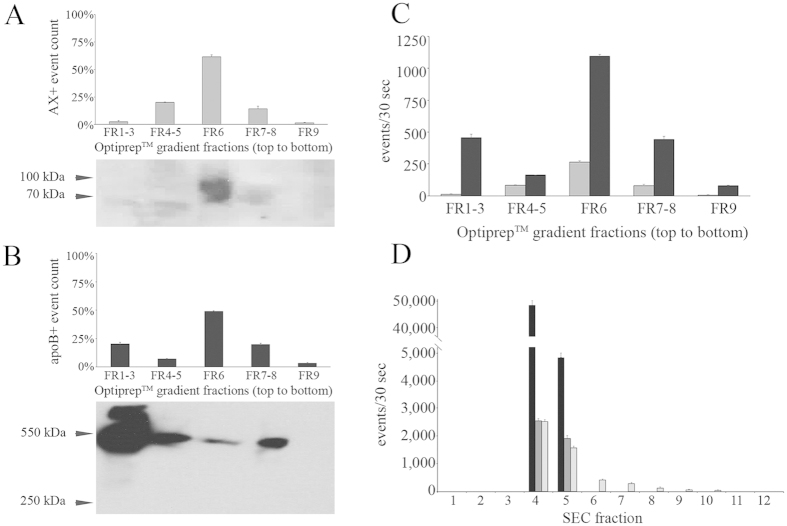
Purification of PLT concentrate-derived MVs with Optiprep^TM^ density gradient UC and SEC. (**A**,**B**) Quantification of FCM data and Western-blotting of PLT concentrate-derived MV fractions purified on an Optiprep^TM^ density gradient (n = 3, mean + SEM). The event number was detected within the MV-gate. MVs were detected by AX (FCM) and CD63 (Western blotting) (**A**) and lipoproteins by apoB (550 kDa) (**B**). Of note, Western blotting only shows one of the analyzed samples while the FCM shows the average ± SEM of 3 measurements. (**C**) Comparison of the apoB-positive events (black bars) and the AX-positives (gray bars) (FCM, n = 2, mean + SEM). (**D**) SEC purification of MVs isolated from PLT concentrates, fractions analyzed by FCM. (apoB: black; AX: gray; CD41a: light gray bars, n = 2, mean + SEM) Note that the apoB-positivity was co-purified with the EV markers.

We found apoB-positivity in MVs isolated from fasting PFP (Fig. 3a,b), so we decided to investigate whether lipoproteins also co-purified with PLT concentrate-derived MVs. For purification we used an Optiprep^TM^ gradient (Fig. 4a,b). Using FCM and Western blotting, AX and CD63 labelling indicated that MVs were enriched in the 6th fraction of the gradient (Fig. 4a) (1.05–1.10 g/mL, Supplementary Fig. S6). Surprisingly, apoB-positive events were detectable in almost all fractions (Fig. 4b). Western blot analysis proved that this apoB-positivity was due to the presence apoB100 (550 kDa) suggesting that the co-purified particles were mostly LDLs, not chylomicrons (bearing apoB48, 260 kDa). Even in the most MV-enriched fraction (FR6), apoB-positive events outnumbered the AX-positive ones up to 5× (Fig. 4c). Next, we tested whether SEC resulted in pure PLT-MVs (Fig. 4d). The third fraction that contained the most pure population of EVs according to the manufacturer contained still high amount of apoB-positive particles measured by FCM.

To further verify the presence of LDL, we carried out MS analysis of MVs isolated from pre- and postprandial blood plasma samples. We identified apoB100 protein in both types of samples among the 10 most abundant proteins, indicating the presence of LDL in these isolated MV samples (Supplementary Table S2).

### LDL co-purifies not only with MVs, but also with EXOs from blood and PLT concentrates

**Figure 5:**
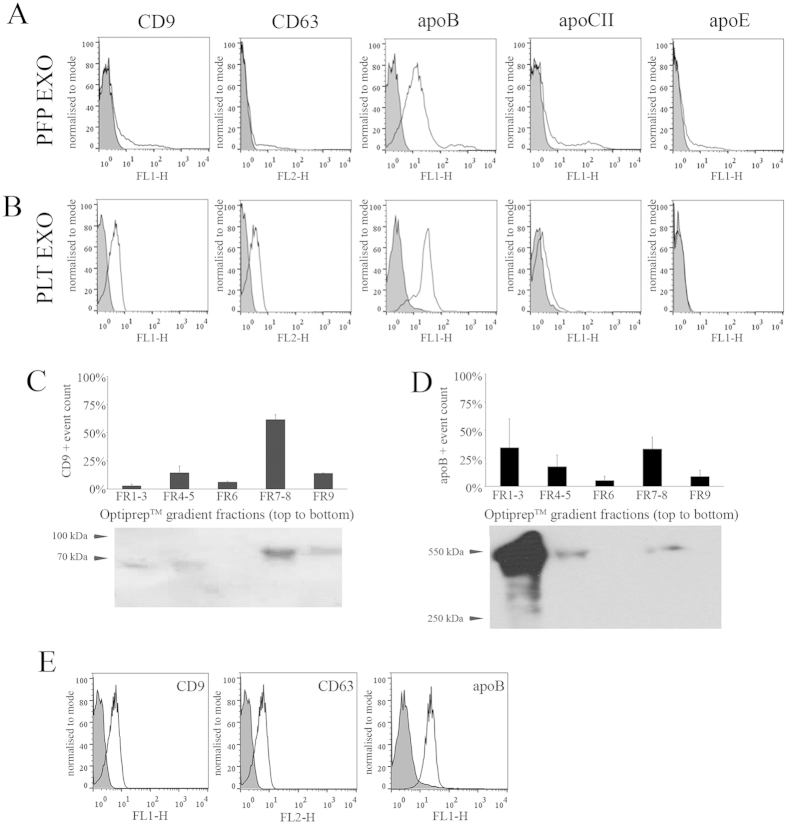
Analysis of apoB-positivity in blood plasma and PLT concentrate-derived EXOs. (**A**,**B**) FCM detection of the indicated markers in EXOs conjugated onto latex beads. The EXOs were isolated from fasting PFP (**A**) or PLT concentrate (**B**) by differential UC and gravity size filtration (gray histograms: blocked beads incubated with antibody, empty histograms: EXO sample). (**C**) EXOs from fasting PFP purified on an Optiprep^TM^ density-gradient. Distribution of CD9 positive events was determined by FCM (upper panel, n = 3, mean + SEM) and CD63 positivity of fractions was determined by Western blotting (lower panel). Of note, Western blotting only shows one of the analyzed samples while the FCM shows the average ± SEM of 3 measurements. (**D**) The distribution of apoB-positive events determined by FCM (upper panel, n = 3) and by Western blotting (lower panel) from the same samples as in (**C**). (**E**) PLT-derived EXOs were also purified on a density-gradient. The EXO containing FR7-8 was analyzed by FCM for CD9-, CD63- and apoB-positivity (gray histograms: blocked beads incubated with antibody, empty histograms: EXO sample).

Since we detected the co-purification of lipoproteins with MVs, next we tested whether they co-purified with EXOs as well. EXOs isolated by differential UC and gravity size filtration from fasting PFP (Fig. 5a) and PLT concentrate (Fig. 5b), were bound onto latex beads for FCM analysis. As expected, they stained for EXO markers such as CD9 and CD63. Importantly, they also showed very high apoB-positivity and a weaker staining for apoCII and ApoE. We also analyzed EXOs isolated form postprandial PFPs. We found that even in fasting condition apoB-positive events covered the surface of beads predominantly (>95% was apoB-positive compared to the <0.5% CD9/CD63/AX-positivity; data not shown). Postprandially we could not detect a significant further increase in the apoB signal. Given the predominant apoB-positivity already present in fasting EXO samples, similarly to the analysis of MVs, in our further purification studies we focused on EXOs derived from fasting PFPs only.

We purified EXOs from fasting PFP samples on an Optiprep^TM^ density gradient and analyzed the fractions by FCM and Western blotting. We found that EXOs were enriched in FR7-8 (1.12–1.18 g/mL, Supplementary Fig. S6) as identified by the presence of CD9 and CD63 (Fig. 5c). Lipoproteins were detected mostly in the top fractions of the gradient, however, a high amount was also found in the EXO-containing FR7-8 (Fig. 5d). Western blotting revealed that the lipoprotein found in the EXO-containing fractions was LDL, since we detected apoB100, but not apoB48. Even when we purified EXOs from PLT concentrates on an Optiprep^TM^ gradient, the purified particles stained intensively for apoB when analyzed by FCM. This suggests that EV preparations isolated from blood or PLT concentrates might contain significant amount of LDL (Fig. 5e).

### LDL overlaps with the size range of EVs and binds to EVs *in vitro*

**Figure 6:**
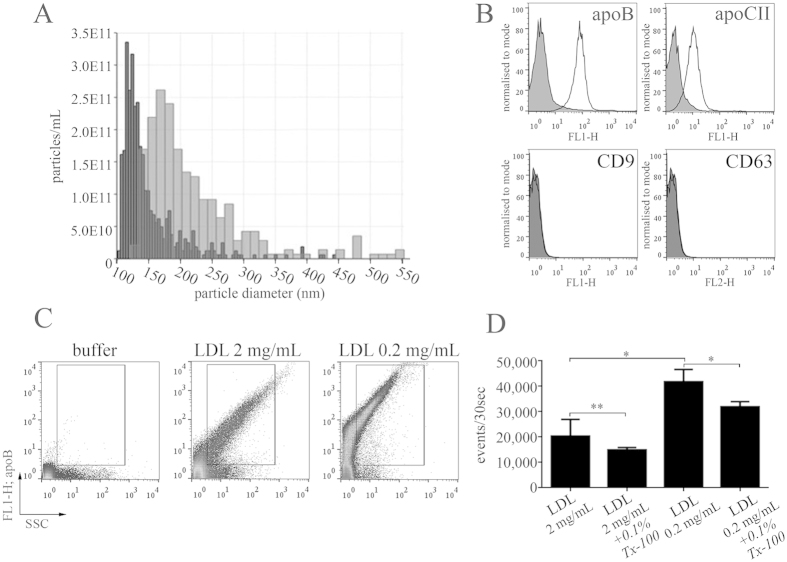
LDL mimics EVs during TRPS and FCM. (**A**) Commercially available human LDL analyzed by TRPS. Note that particles with different sizes were detected within the MV size range (100–800 nm) (measured on NP150 and NP300 nanopore membranes, black and gray bars, respectively). (**B**) FCM analysis of LDL particles conjugated to latex beads. Commercial LDL stained for apoB and apoCII, but not for the EV markers CD9 and CD63 (black filled histograms: antibody control, empty histograms: LDL). (**C**) LDL detection by FCM without bead conjugation at different dilutions. Note that the measured event number increases with the dilution, suggesting a swarm effect. (**D**) The signal obtained from commercial LDL is also partially sensitive to 0.1% Tx-100 at a physiological concentration (2 mg/mL) and in the 10 × diluted sample (0.2 mg/mL) as well (*P < 0.05, **P < 0.01, Mann-Whitney test).

To test the hypothesis that the apoB100 positive particles represent LDL, purified human LDL from a commercial source (both from Sigma-Aldrich and Merck) was assessed by TRPS as shown in Fig. 5a. Strikingly, the sizes of particles ranged from 100 to 500 nm possibly suggesting aggregation of the LDL particles (Fig. 6a). TRPS analysis of LDL at 2 mg/mL concentration (similar to that of blood plasma^25^,^26^) resulted in a two orders of magnitude difference in the detected particle number between NP100 and NP200 (Supplementary Fig. S7). The concentration at the average diameter of 110 nm was 1.0E13/mL, while the estimated particle number of LDL in human plasma is reported to be approximately 1.0E14/mL^26^, suggesting that we still only see the tip of the iceberg. We also analyzed LDL particles conjugated onto latex beads by FCM, and detected apoB and apoCII positivity on the bead surface (Fig. 6b). As expected, in the case of LDL no signal was detected for the EV markers CD9 and CD63 (Fig. 6b). We next asked whether LDL particles were also detectable by FCM without bead conjugation, and surprisingly, LDL at a concentration of 2 mg/mL^25^,^26^ was readily detected by FCM. We demonstrated that the signal was caused by a swarm effect of LDL particles. Using serial dilution of commercial LDL we have shown that the detected event number increased in the diluted LDL sample (Fig. 6c,d). A similar swarm effect has been reported to influence the FCM detection of EVs^27^. Indeed, we also demonstrated this swarm effect by testing a PFP sample (Supplementary Fig. S8). Furthermore, we showed that LDL at the above physiological concentration shared side-scattering properties with MVs isolated from human blood plasma both in fasting and in postprandial states (Supplementary Fig. S9). We also analyzed the detergent sensitivity of commercial LDL and found that LDL was partially sensitive to 0.1% Tx-100, possibly due to the disruption of lipoprotein aggregates by the detergent (Fig. 6d).

**Figure 7:**
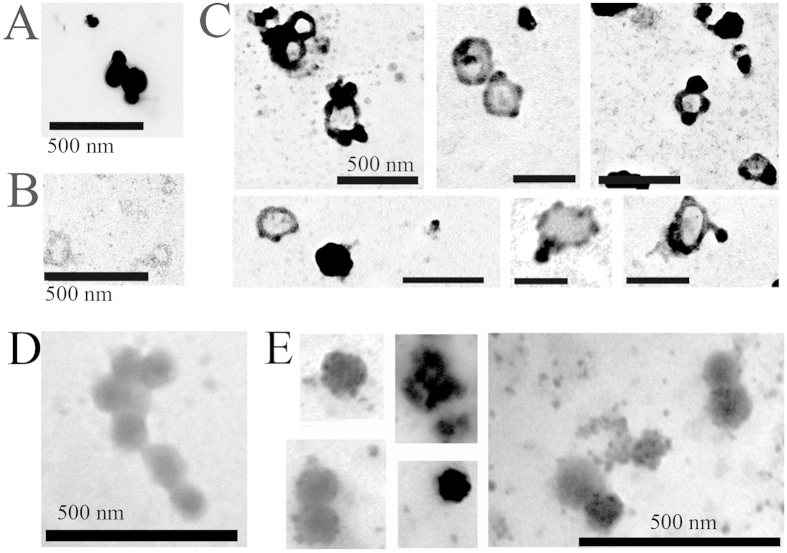
LDL binds onto isolated MVs and EXOs *in vitro*. (**A,B**) TEM analysis of commercial LDL (**A**) and cell line-derived MVs (**B**) using the “osmification-on-grid” method. LDL particles appear as highly electron-dense, round structures and show aggregation (**A**) (scale bars: 500 nm). MVs isolated from the conditioned media of a cell line had vesicular morphology (**B**) (scale bars: 500 nm). (**C**) TEM images obtained after *in vitro* mixing of commercial LDL with cell-derived MVs for 1 h at room temperature (scale bar: 500 nm). Electron-dense LDL was observed to bind extensively onto MVs, to cover their surfaces, and to form aggregates in the size range of the vesicles. Note that almost all MVs were covered with LDL particles to various extents. (**D**) TEM picture of cell line-derived EXOs (scale bar: 500 nm). (**E**) Images of cell line-derived EXOs *in vitro* mixed with commercial LDL. Note that the surfaces of the EXOs are covered with LDL particles. The electron dense particles in the size range of EXOs represent LDL aggregates (scale bar: 500 nm).

Next, we investigated further the capacity of LDL to associate with EVs. Using the “osmification-on-grid” approach, TEM revealed that commercial LDL was highly similar to the structures that we found in our PFPs previously (highly electron-dense round particles) (Fig. 7a). In contrast, MVs isolated from a cell line in serum free conditions, showed the typical vesicular morphology (Fig. 7b). Upon *in vitro* mixing LDL at a concentration close to physiological with the cell line-derived MVs, we found that LDL bound extensively onto MVs, covered their surfaces, and formed aggregates within the size range of the vesicles (Fig. 7c). Importantly almost all MVs were covered with lipoproteins to various extents. We also analyzed the LDL-association of cell-line derived EXOs. Upon *in vitro* mixing the EXOs with LDL, we detected the same phenomenon as with the MVs. The LDL particles associated with EXOs, and formed aggregates in the EXO size range as well (Fig. 7d,e). In these experiments we did not perform any additional washing step after mixing LDL with EVs (MVs or EXOs); therefore the association of LDL with EVs was not a result of co-pelleting.

## Discussion

In recent years EV research has not only achieved high visibility but also attracted an increasing interest from various fields of biology and medicine. One of the most exciting aspects of EVs in the blood plasma is that they may serve as biomarkers in different diseases^5^,^6^,^7^,^8^,^9^. Thus, there has been an outstanding interest in blood plasma-derived EVs, however, purification of EVs from blood is hampered by the complexity of this bio fluid. In this work we introduced modifications in techniques currently used for EV analysis. This enabled us to detect for the first time circulating lipoproteins and EVs simultaneously by FCM, Western blotting and TEM.

Our initial finding, the appearance of chylomicrons in postprandial PFP samples, was evidenced by i) the elevated serum triglycerides in samples upon food intake, ii) the increased concentration of particles postprandially and iii) the appearance of highly electron-dense particles on the top of ultracentrifuged PFP. Postprandial triglyceride transportation, floatation behavior upon UC and a characteristic TEM morphology are known features of chylomicrons, which have been reported to fall into the size range of 200–600 nm^14^,^22^,^28^,^29^. Although we found chylomicrons to float abundantly only on the top of postprandial PFP samples, surprisingly, a very high number of particles in fasting samples also stained with an anti-apoB100/B48 antibody by FCM. In blood plasma the vast majority of apoB100 is carried by LDL (~25 nm)^14^, while less than 10% of it is carried by intermediate-density lipoprotein (IDL, ~25–35 nm)^16^ and very low-density lipoprotein (VLDL, ~30–80 nm)^16^ together^30^. The other isoform of apoB, apoB48 is the marker of chylomicrons^31^. Distinguishing the two apoB isoforms was only possible by Western blotting, because commercial antibodies that recognize apoB100 (550 kDa) also react with the truncated form of the protein, apoB48 (260 kDa). Western blot analysis of our samples revealed that EVs purified on a density gradient not only carried EV markers but also the apoB100 molecule, suggesting that the vast majority of co-purified particles were LDLs. This was true for both MVs and EXOs, either isolated from PFP or from PLT concentrates.

As expected, postprandially we could detect chylomicrons by conventional FCM due to their relatively big size. Nevertheless, attention has to be paid to the time of blood sampling, as 1 h postprandially chylomicrons are still hardly detectable. However, their amount increases rapidly thereafter. In contrast to chylomicrons, the concentration of LDL slightly decreases upon food intake^32^. This stands in line with our finding that routine laboratory determination of apoB100 showed a small decrease in postprandial samples (Supplementary Table S1).

LDL particles are too small to be detected individually by FCM^14^. However, the concentration of LDL in blood plasma is approximately 20,000× higher than that of chylomicrons (2 mg/mL vs. 0.1 mg/L)^22^,^31^. This high concentration in blood plasma and their high lipid content enabled their detection by conventional FCM due both to a swarm effect and their increased scattering properties. Even though FCM is not suitable for determining the exact number of LDL particles, we could still gain an insight into their magnitude in blood plasma. Antibodies to apoCII and apoE did not give as prominent staining as anti-apoB. This was in line with the fact that apoB100 constitutes more than 95% of the total protein mass of LDL^33^ and each LDL particle carries a single copy of apoB100 on its surface, while only 10% carries apoE or apoCII^14^. Taken together, while the postprandial increase of apoB-positivity detected in this study was due to the appearance of chylomicrons in blood plasma, the high pre-prandial apoB signal was related to LDL.

Our analysis revealed that lipoprotein particles were not only detectable by various EV assessment methods, but could not be separated from EVs by any of the currently available purification techniques. Importantly, we found that incubation of isolated nascent cell-derived EVs with commercial LDL for 1 h at room temperature at a concentration characteristic for healthy blood plasma^25^ resulted in a dramatic attachment of LDL particles onto the surface of EVs. The above association was probably mediated by the known interaction of LDL with hydrophobic surfaces^34^ and EVs have been reported to expose the hydrophobic core of their phospholipid bilayer because of curvature-related short lived lipid packing defects^35^. The decreased staining for EV markers in postprandial state might be explained by hindrance of vesicle surfaces by the attached lipoproteins. This may at least partially explain the presence of the puzzling AX-negative and/or EV-marker negative “orphan” vesicles in bio fluids^36^.

How could we miss lipoprotein particles until now? Why did not we detect them before? Our data suggest that lipoproteins are present in such high numbers that we need to use substantially more antibodies than usual in order to detect them (Supplementary Fig. S3). Detection of apoB100 with Western blotting is difficult due to its very high molecular weight. This is why we chose to use agarose gels to visualize the apoB100 band at 550 kDa by Western blotting. Furthermore, EV suspension on-grid analysis by TEM usually involves the use of phosphotungstic acid or uranyl acetate, but not OsO_4_^4^,^37^,^38^. Here we used osmification of lipoprotein-containing droplets prior to TEM, and this enabled us to visualize lipoprotein particles in our vesicle suspensions isolated from blood plasma as highly electron-dense structures.

HDL has been reported to co-purify with EXOs due to its floatation density (1.063–1.21 g/mL) similar to that of EXOs^20^. Our unexpected finding is that LDL which has a floatation density (<1.06 g/mL)^14^ lower than that of either EXOs or HDL particles, is present both in MV and EXO preparations (even after density gradient ultracentrifugation). Moreover, it was also present in EV preparations derived from PLT concentrates. Our data are supported by a previous MS study that reported the presence of apoA-I, apoB and apoE in blood plasma-derived EXO preparations^39^. Similarly, our MS analysis of fasting and postprandial plasma-derived MVs also showed the presence of the above apolipoproteins (Supplementary Table S2). Additionally, looking at this question from an entirely different perspective, a proteomic analysis of purified VLDL and LDL has shown the presence of CD14, protein S100-A8, HLA class I molecules and LDL-receptor (proteins known to be associated with EVs)^40^.

The most important practical implication of our data is that LDL resembles circulating EVs (both EXOs and MVs) and with the currently used isolation methods cannot be separated from them. Importantly, the size of LDL particles is highly variable (Fig. 6a) most likely due to aggregation or fusion of these particles. Both PFP-derived apoB-positive particles and purified LDL were partially sensitive to detergent lysis. Thus, detergent sensitive events could be either lipoprotein aggregates or MV-lipoprotein complexes. Given the presence of at least one order of magnitude higher concentration of LDL particles (Fig. 2b) than EVs, detection of blood plasma-derived EVs based on particle enumeration (by nanoparticle tracking analysis or TRPS) might strongly overestimate EV numbers. Furthermore, studies conducted with blood-derived EVs should consider the presence of co-purified LDL. Thus, our data not only serve the integrity of circulating EV detection but may also provide new insights into the pathological processes in which LDL or EVs have been implicated earlier.

## Methods

### Blood donors

Venous blood was collected from 14 healthy donors (6 males and 8 females, mean age ±SEM: 27.14 ± 1.77 years, range: 25–30 years) in fasting state and 4 h postprandially. Subjects consumed a high-fat standardized breakfast (65% fatty acids, 33.4% protein and 1.6% carbohydrates). During the entire investigation period, we followed the guidelines and regulations of the Helsinki Declaration in 1975, and the experiments were approved by the Hungarian Scientific and Research Ethics Committee; all tested individuals signed an informed consent form.

### Blood collection and platelet free plasma (PFP) preparation

We followed a previously described protocol^11^,^24^ and the guidelines of ISTH on blood sampling and handling for MV analysis^41^. Briefly, blood was collected into acid-citrate-dextrose (ACD-A) tubes (Greiner Bio-One) in order to prevent *in vitro* release of platelet-derived EVs^11^. Blood was centrifuged 2 × 15 min at 2,500 g. Platelet-free plasma (PFP) was aliquoted, snap frozen in liquid nitrogen, and stored at −80 °C until analysis.

### Routine laboratory measurements

Routine laboratory determination of serum triglycerides, total cholesterol, LDL cholesterol, apoA1 and apoB100 in fasting and postprandial states was performed in the Central Laboratory at Semmelweis University, Budapest, Hungary with a Beckman Coulter AU680 instrument.

### EV isolation from PFP

EVs were isolated as described previously^11^,^19^,^24^. Briefly, PFPs were diluted 1:1 in phosphate-buffered saline (PBS)/annexin binding buffer (ABB: 10 mmol/L HEPES, 140 mmol/L NaCl; 0.25 mmol/L CaCl_2_; pH: 7.4–7.5), followed by gravity-driven filtration through a 0.8 μm filter (Whatman). The filtrate was centrifuged at 20,500 g, 16 °C for 60 min in a microcentrifuge. The resulting MV pellet was resuspended in 50 μL buffer and was analyzed by flow cytometry (FCM). The supernatant was gravity filtered through a 0.2 μm filter (Sartorius), and then EXOs were pelleted by ultracentrifugation (UC) at 100,000 g, 4 °C, for 90 min (OptimaMAX-XP, MLA-55 rotor, Beckman Coulter Inc).

In some experiments, Optiprep^TM^-density UC was performed with both MV and EXO pellets as described previously^42^. Briefly, 5, 10, 20 and 40 w/v% Optiprep^TM^ (Sigma-Aldrich) layers were used starting with 40 w/v% at the bottom. The sample was layered onto the top, and UC was performed at 100,000 g, 4 °C for 20 h (Beckman Coulter OptimaMAX-XP, MLS-50 rotor). Fractions were collected from top to bottom. Each fraction was diluted with PBS and centrifuged at 100,000 g (MLA-55 rotor) or in the case of MVs, at 20,500 g. The pelleted fractions were either resuspended for FCM or lysed for Western blotting.

### EV isolation from cell line and human platelet concentrates

The 5/4E8 Th1 T hybridoma cell line generated by our group^43^ was cultured as described previously^44^. For EV production, cells were grown for 24 h in serum-free medium. MV and EXO isolation was performed as described previously^23^,^24^.

For isolation of platelets (PLT) and PLT-derived EVs, human PLT concentrate was purchased from the Hungarian National Transfusion Service, Budapest, Hungary on the day of expiry. For PLT-derived EV isolation we used a recently described protocol^24^,^45^.

### Flow cytometry (FCM)

PFP samples were analyzed using a FACSCalibur flow cytometer (BD Biosciences). Instrument settings and gates were adopted from previous works^11^,^19^,^23^,^24^. The MV gate was set up by using Megamix^TM^ Beads (Biocytex). Gating was optimized using 1 μm silica beads with a refractory index similar to that of EVs (Fig. 1a and Supplementary Fig. S2). All samples were measured at low flow rate for 30 sec, the variations in the flow rate of the instrument were assessed by using 1 μm polystyrene calibration beads with a constant concentration. The flow rate variation was less than 2.5% (21.11 ± 0.52 μL/min) in all performed experiments. MV markers were determined in 10 μL PFP using 1 μL of the following reagents in 60 μL total volume: annexin-V-PE (AX), anti-CD41a-APC (both from BD Biosciences, anti-CD41a clone: HIP8), anti-CD9-FITC, anti-CD63-PE (both from Sigma–Aldrich, clones MEM-61 and MEM-259, respectively). Apolipoprotein B (apoB) staining of PFPs (from 0.2 to 20 μL; Supplementary Fig. S3, lower panel) was performed using 4 μL of a goat polyclonal anti-human-apoB100/48-FITC antibody (Mybiosource) in a total volume of 60 μL. Self-aggregates of this antibody were removed prior to staining by centrifuging a 10-fold diluted antibody solution at 20,500 g for 1 h. Only the aggregate-free supernatant was used for subsequent staining (Supplementary Fig. S10). For comparable analysis, data of 10 μL anti-apoB-stained PFP is presented. Stainings for apoCII and apoE were performed using 4 μL of goat polyclonal anti-apoCII-FITC antibody (Abcam) or 4 μL of anti-apoE-FITC antibody (Novus Biologicals, clone WUE-4). All samples were immune labeled at room temperature in the dark for 30 min. After staining, the unbound antibodies were removed by diluting the samples, and pelleting the EVs at 20,500 g (Supplementary Fig. S11). To confirm the presence of EVs, we applied detergent lysis and only events that disappeared in the presence of 0.1% Triton X-100 (Tx-100) were considered vesicles^19^,^23^.

MVs were isolated from 500 μL PFP as described previously^19^,^23^, resuspended in 20 μL PBS and the same stainings as for PFPs were performed.

Bead-bound EXOs were analyzed by FCM as described previously^24^. Briefly, they were conjugated onto aldehyde/sulfate latex beads (Life Technologies), blocked with 100 mmol/L glycine and 1 w/v% bovine serum albumin (BSA) in PBS overnight at 4 °C. After removing the blocking solution (4,000 g; 15 min), the pellet was resuspended in PBS for immunostaining.

Flow cytometry data was analyzed with the FlowJo 10.0.8 software.

### Tunable Resistive Pulse Sensing (TRPS)

PFP samples and EV preparations were analyzed by TRPS using a qNano instrument (IZON Sciences Ltd.) as described previously^11^,^23^,^24^,^46^. Briefly, NP100, NP150, NP200, NP300, NP400 and NP800 nanopore membranes were used to measure the samples in serial dilution. We counted at least 500 events/sample (in case of very low particle concentration, we measured for 5 min). Calibration was performed using calibration beads with a defined concentration, provided by the manufacturer (IZON).

### Transmission electron microscopy (TEM) of EVs and LDL

EV-pellets and EV-suspensions were visualized using two different approaches. EV-pellets were processed as described previously^6^,^11^,^23^,^24^,^44^,^46^. Briefly, pellets were fixed with 4% paraformaldehyde, postfixed in 1% osmium tetroxide (OsO_4_) and after rinsing, pellets were dehydrated by a series of increasing ethanol concentrations, including block staining with 1% uranyl-acetate in 50% ethanol for 30 min, and embedded in Taab 812 (Taab). Ultrathin sections were analyzed with a Hitachi 7100 electron microscope equipped by Veleta, a 2000 × 2000 MegaPixel side-mounted TEM CCD camera (Olympus).

EV suspensions were analyzed by a rapid “osmification on grid” approach^22^,^28^. Identical volumes of suspensions of freshly isolated MVs and buffered 1% OsO_4_ were mixed and placed onto Formvar coated grids (10–30 min). Three brief washes in water (3 × 5 min) followed, next contrast staining with 1% uranyl-acetate in 50% alcohol for 15 min, and finally water again (3 × 5 min). The grids were air-dried and analysed by TEM as described above. In the *in vitro* mixing experiments of LDL and EXOs only OsO_4_ was used for visualization of samples on the grid.

### Size exclusion chromatography (SEC)

In order to eliminate the lipoproteins present in our samples, PFP samples (both from fasting and postprandial individuals) as well as MVs isolated from PLT concentrate were subjected to SEC^47^. We used the qEV^TM^ column of IZON, according to the instructions of the manufacturer. Briefly, the column was rinsed with filtered PBS, and then either 0.5 mL of PFP or PLT-MVs in PBS were layered onto the top. After collection of a 3 mL void volume, 1 mL EV-containing fraction and further 8 × 1 mL fractions were collected. The fractions from plasma were immediately measured by TRPS, whereas the PLT-MVs were pelleted at 20,500 g and analyzed by FCM.

### Western blot

Five percent agarose (Sigma-Aldrich) was dissolved in a buffer containing 25 mmol/L Tris base, 190 mmol/L glycine and 0.1% sodium-duodecyl-sulfate (SDS), and a horizontal gel was prepared. Optiprep^TM^ gradient fractions with identical volumes were pelleted, resuspended in lysis buffer, and electrophoresed in agarose-SDS gel at constant 100 V for 5 h. Proteins were blotted to PVDF membranes (Bio-Rad). Membranes were blocked in 1% BSA in TBS-Tween, and were incubated overnight at 4 °C in the same buffer with rabbit polyclonal anti-human-apoB100/48 (Novus Biologicals), or with rabbit monoclonal anti-human-CD63 (Santa Cruz Biotech). Secondary immunostaining was performed with a goat anti-rabbit-immunoglobulin-HRP conjugate (Abcam). Chemiluminescent signals were detected with the Pierce ECL Western blotting substrate (Thermo Fisher Scientific). Blots are shown in Supplementary Figs S12 and S13.

### Mass spectrometry (MS)

EV pellets were resuspended in 20 μL water and proteins were extracted using repeated freeze-thaw cycles^44^. Proteins were digested as described previously^48^. The peptide digest was analyzed using a Bruker Maxis II Q-TOF instrument with CaptiveSpray nanoBooster ionization source. Peptides were separated online using a 25 cm Acclaim Pepmap RSLC nano HPLC column on the Dionex Ultimate 3000 Nano LC System. Data were evaluated with ProteinScape 3.0 software, using Mascot search engine.

### Statistics and data analysis

Statistical analysis was performed with GraphPad Prism v.6 software. For comparison of fasting and postprandial samples, we used paired t-test or Wilcoxon matched-pairs signed rank test. Samples were tested for normality with the D’Agostino-Pearson normality test. For comparison of several groups 1-way ANOVA was used followed by Dunnetts post-hoc test. (*P < 0.05, **P < 0.01 and ***P < 0.001). Error bars indicate standard error of the mean (SEM).

Images were edited with the Adobe Photoshop CS5 software.

## Additional Information

**How to cite this article**: Sódar, B. W. *et al.* Low-density lipoprotein mimics blood plasma-derived exosomes and microvesicles during isolation and detection. *Sci. Rep.* **6**, 24316; doi: 10.1038/srep24316 (2016).

## Supplementary Material

Supplementary Information
