# Supplementary material for: Low-density lipoprotein mimics blood plasma-derived exosomes and microvesicles during isolation and detection
Source: Sci Rep. 2016 Apr 18;6:24316. doi: 10.1038/srep24316 (PMC4834552; doi:10.1038/srep24316)
Supplement: Supplementary Information [file srep24316-s1.doc]

***SUPPLEMENTARY MATERIAL***

**Low-density lipoprotein mimics blood plasma-derived exosomes and microvesicles during isolation and detection**

Barbara W Sódar^1^, Ágnes Kittel^2^, Krisztina Pálóczi^1^, Krisztina V Vukman^1^, Xabier Osteikoetxea^1^,
 Katalin Szabó-Taylor^1^, Andrea Németh^1^, Beáta Sperlágh^2^, Tamás Baranyai^3^, Zoltán Giricz^3^, Zoltán Wiener^1^, Lilla Turiák^4^, László Drahos^4^, Éva Pállinger^1^, Károly Vékey^4^, Péter Ferdinandy^3^, András Falus^1^ and Edit Irén Buzás^1#^

^1^ Department of Genetics, Cell- and Immunobiology, Semmelweis University, Budapest, 1089, Hungary

^2^ Institute of Experimental Medicine, Hungarian Academy of Sciences, Budapest, 1083, Hungary

^3^ Department of Pharmacology and Pharmacotherapy, Semmelweis University, Budapest, 1089, Hungary

^4^ Research Centre for Natural Sciences, Hungarian Academy of Sciences, Budapest, 1117, Hungary

^#^ corresponding author:

Prof. Edit I Buzás

1089, Budapest, Nagyvárad tér 4, 7^th^ floor, HUNGARY

[edit.buzas@gmail.com](mailto:edit.buzas@gmail.com)

Tel: +36-1-2102929

Fax: +36-1-3036968

*Supplementary Figure S1*

*
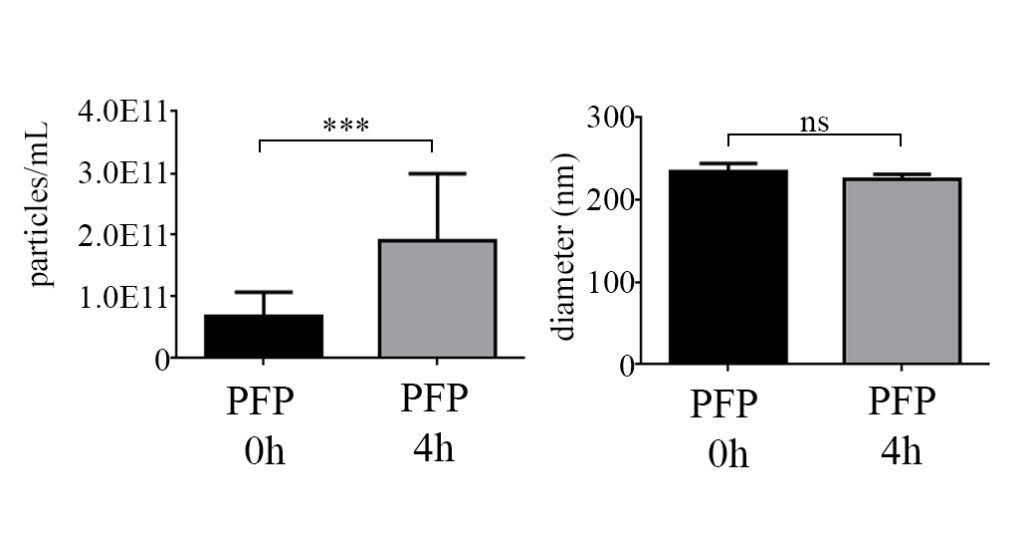
*

Supplementary Fig S1.

Fasting and 4 h postprandial PFP samples analysed with TRPS using NP200. (***P: 0.001, n=5, Wilcoxon matched-pairs signed rank test)

*Supplementary Figure S2*

**
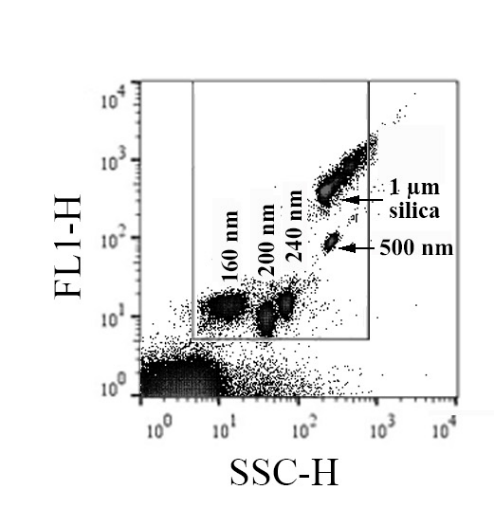
**

Supplementary Fig S2.

Fluorescence-based gating using fluorescent reference beads.

*Supplementary Figure S3*

*
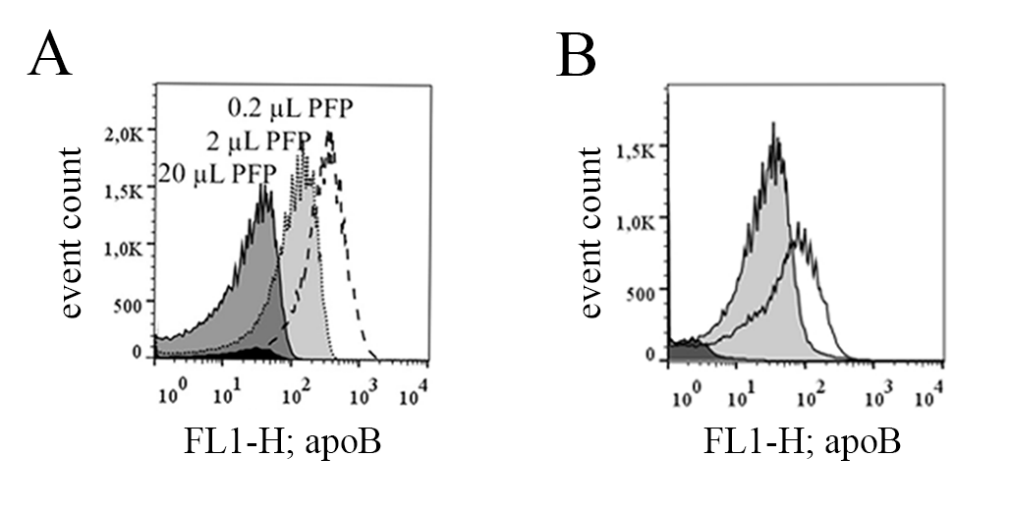
*

Supplementary Fig S3. Reduction of fluorescence intensity when the same amount of anti-apoB antibody (4 µL) is used to stain an increasing number of events.

S3A: An increasing amount of fasting PFP (from 0.2 µL to 20 µL) was stained with a constant 4 µL of anti-apoB antibody. Note the decrease in the detected fluorescence intensity (black histogram: antibody in buffer, other histograms: indicated amounts of PFP samples).

S3B: A representative subject of the 9 analyzed donors used for FCM analysis of MVs isolated form 500 µL PFP. Note that postprandially the amount of antibody (4 µL) which was optimized for staining 10 µL PFP and fasting MVs, is not sufficient to label the whole postprandial population (dark gray histogram: antibody in buffer, empty histogram: fasting MVs, light gray histogram: postprandial MVs).

*Supplementary Figure S4*

*
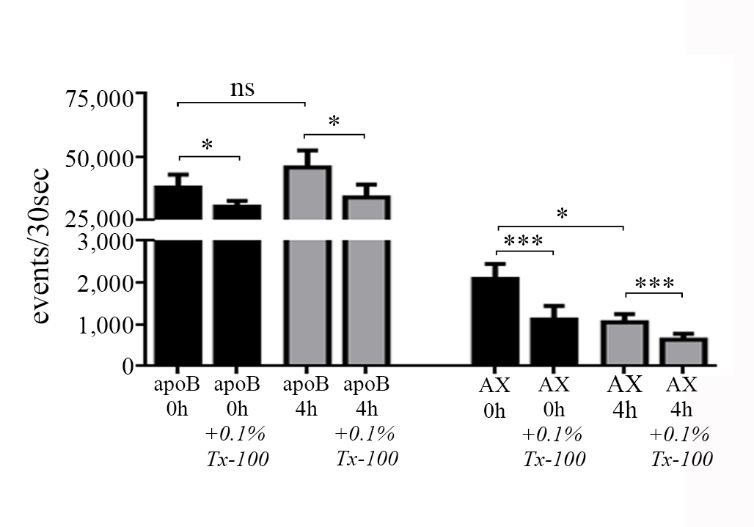
*

Supplementary Fig S4. The detergent sensitivity of MVs isolated from fasting (black bars) and postprandial (gray bars) PFPs stained for apoB and AX (n=9, mean+SEM, *P<0.05, ***P<0.001, Wilcoxon matched-pairs signed rank test).

*Supplementary Figure S5*

*
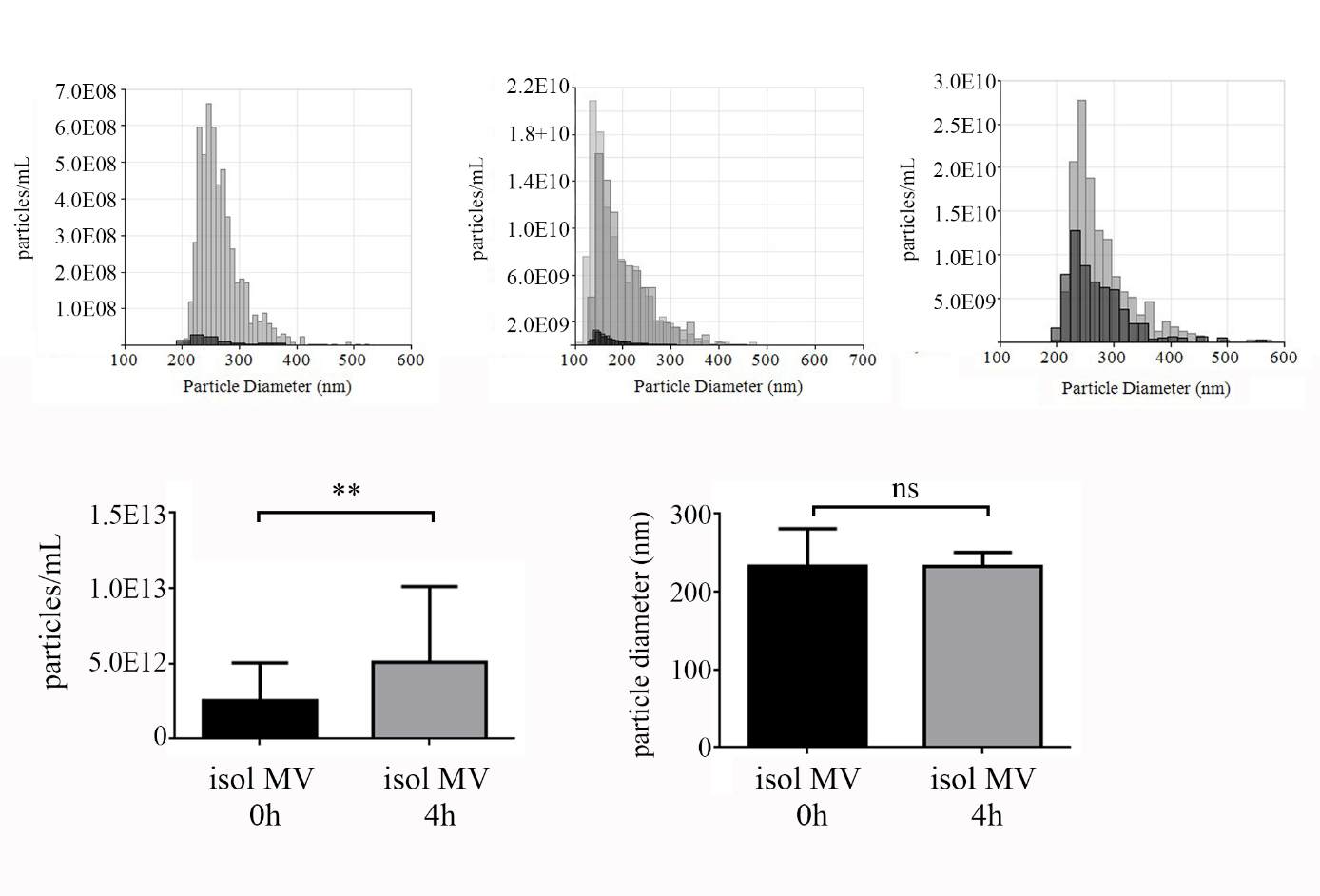
*

Supplementary Fig S5. TRPS analysis of MVs isolated from fasting and postprandial PFPs.

Upper panel: increase in the concentration of particles in 3 different isolated MV samples (black bars: fasting, gray bars: postprandial MVs).

Lower panel: Difference between the fasting and the postprandially isolated MVs (**P: 0.0078; n=8, Wilcoxon matched-pairs signed rank test). The number of isolated particles increased upon food intake, while the mean particle diameter was not affected.

*Supplementary Figure S6*

**
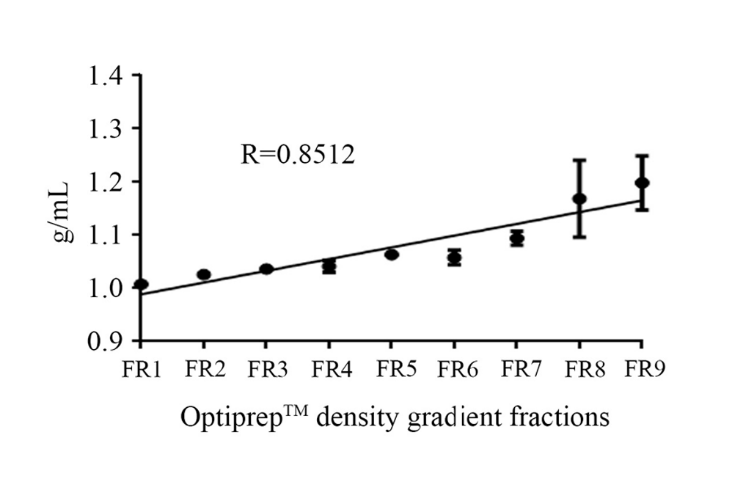
**

Supplementary Fig S6. Representative gravimetric data of Optiprep^TM^ density gradients. EXOs found in FR7-8 floated at a higher density (approx. 1.10-1.18 g/mL) compared to MVs mostly found in FR6 (approx. 1.03-1.05 g/mL).

*Supplementary Figure S7*


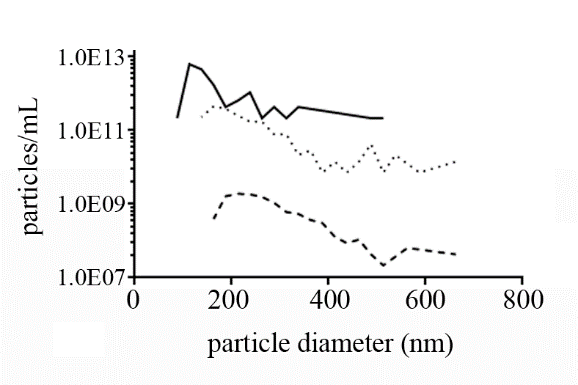


Supplementary Fig S7. Commercial LDL analysed by TRPS. Note that with reducing the pore size the sensitivity increases, and LDL can overlap with both the MV and the EXO size range (dashed line: NP300, dotted line: NP200, continuous line: NP100).

*Supplementary Figure S8*

**
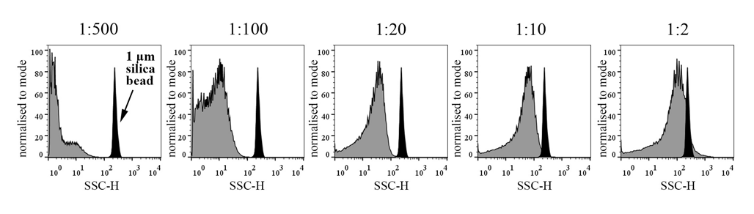
**

Supplementary Fig S8. The impact swarm effect had on size estimation based on SSC. Black histogram represents silica beads with a diameter of 1 µm. Fasting unstained PFP was measured by FCM at different dilutions. Particle size estimation based on SSC was highly altered with increasing concentration.

*Supplementary Figure S9*


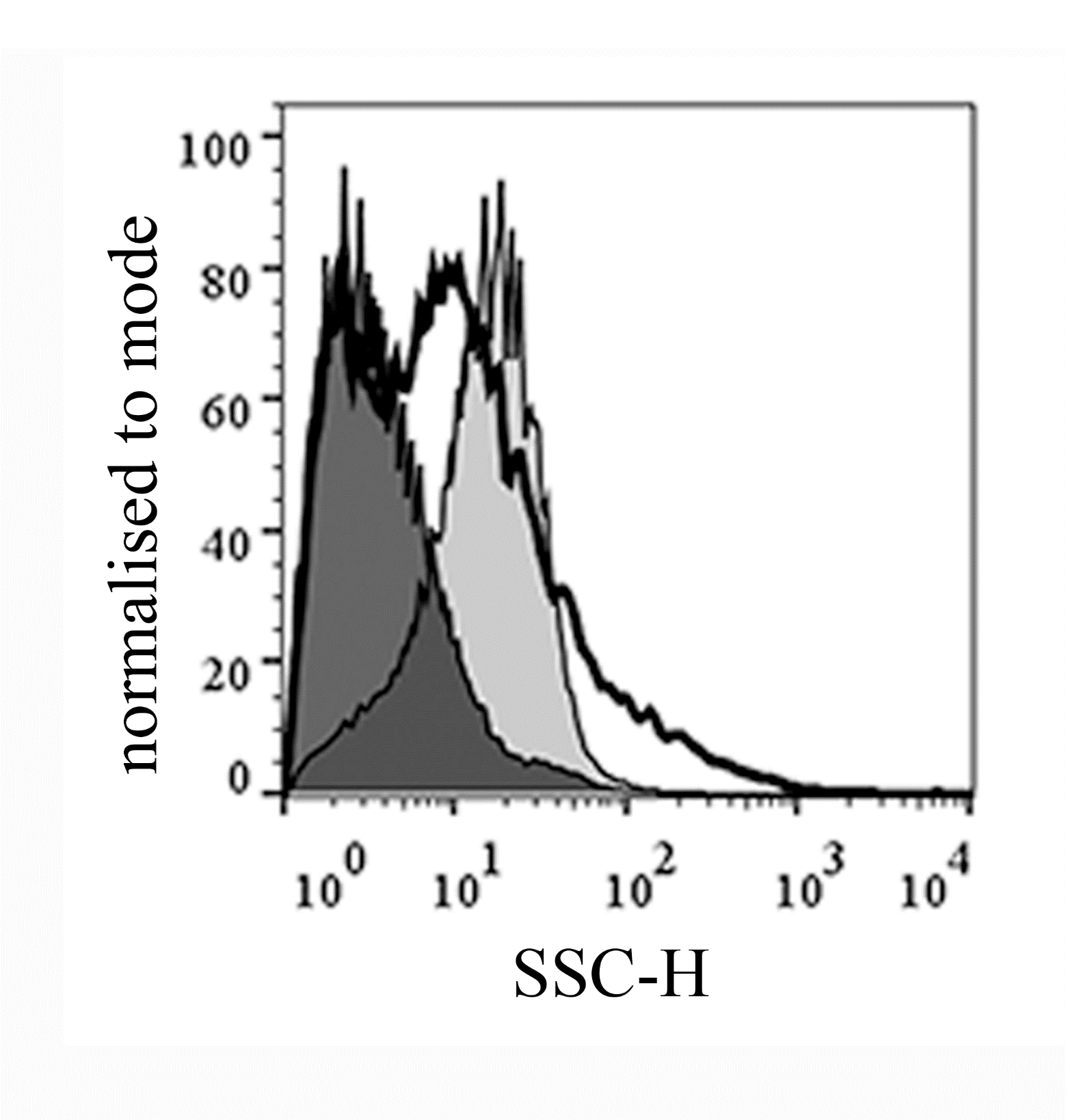


Supplementary Fig S9. Comparison of side scattering properties of commercial LDL and MVs (the latter isolated from fasting and postprandial PFPs) (LDL: empty histogram, postprandial MVs: light gray histogram, fasting MVs: dark gray histogram).

*Supplementary Figure S10*

*
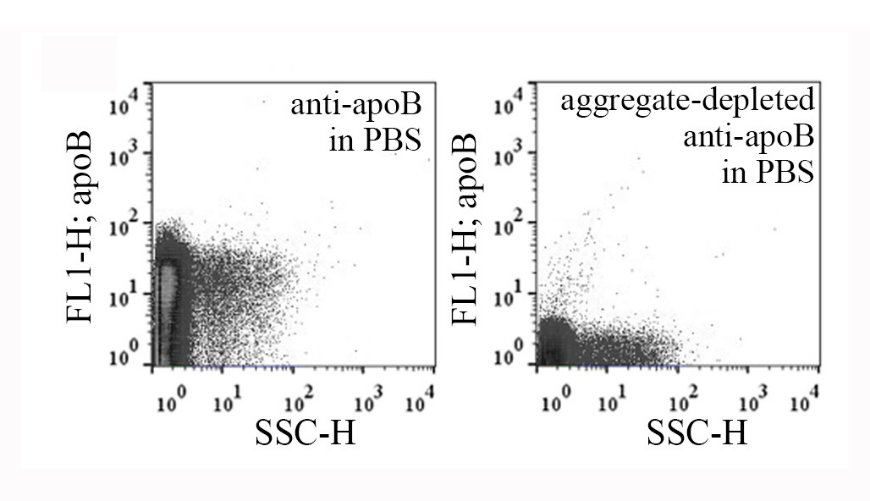
*

Supplementary Fig S10. demonstrates the effects of removing (20,500g 1 h) self-aggregates of the anti-apoB antibody, thus reducing background noise prior to apoB staining for flow cytometry.

*Supplementary Figure S11*

*
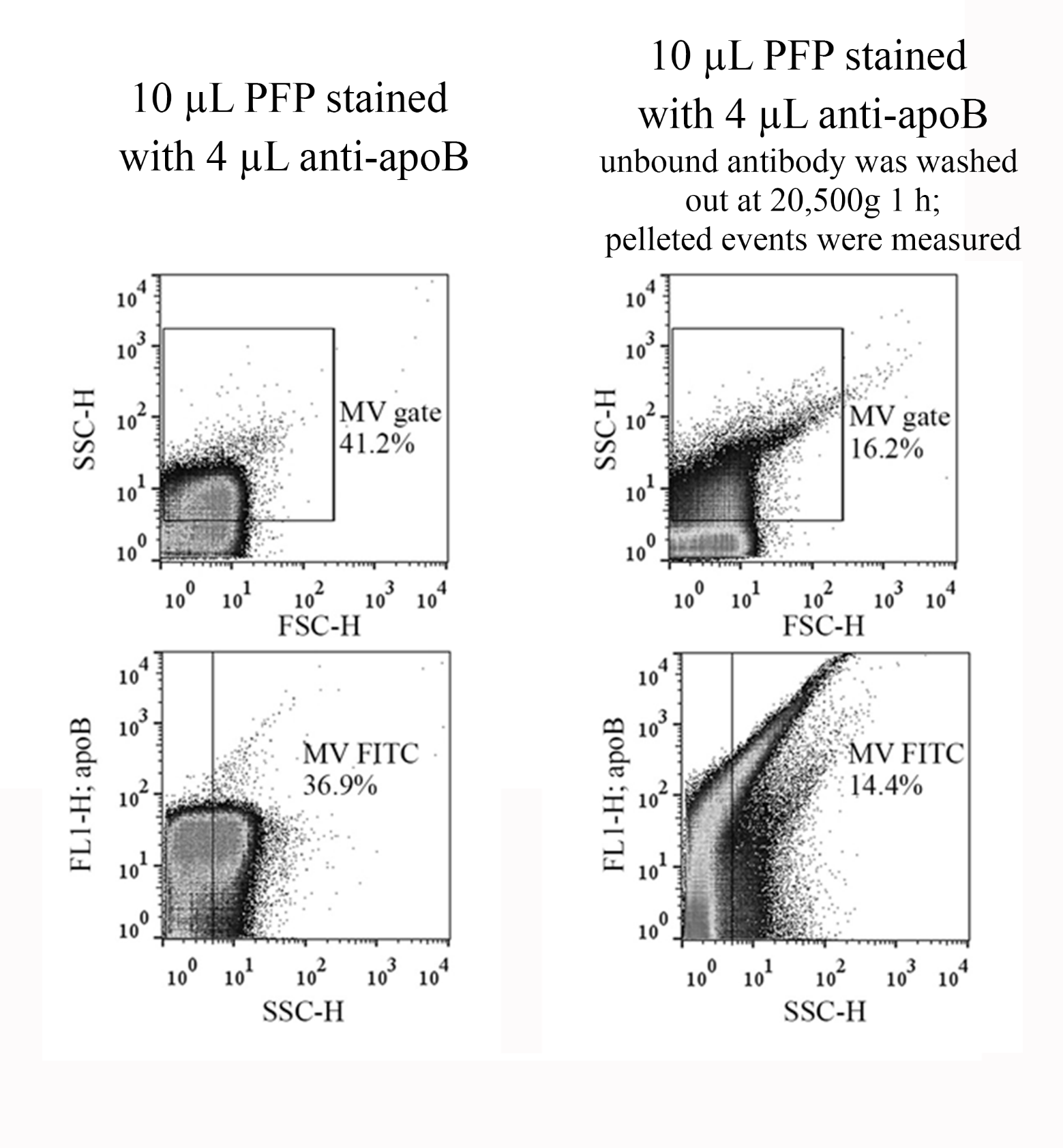
*

Supplementary Fig S11. Flow cytometry of fasting PFP samples. After apoB staining, samples were measured directly (left panel) or the excess of anti-apoB-antibody was removed by centrifugation after the staining procedure (20,500g 1 h) (right panel). Note that approx. 2/3 of the apoB-positive events was not pelleted.

*Supplementary Figure S12*


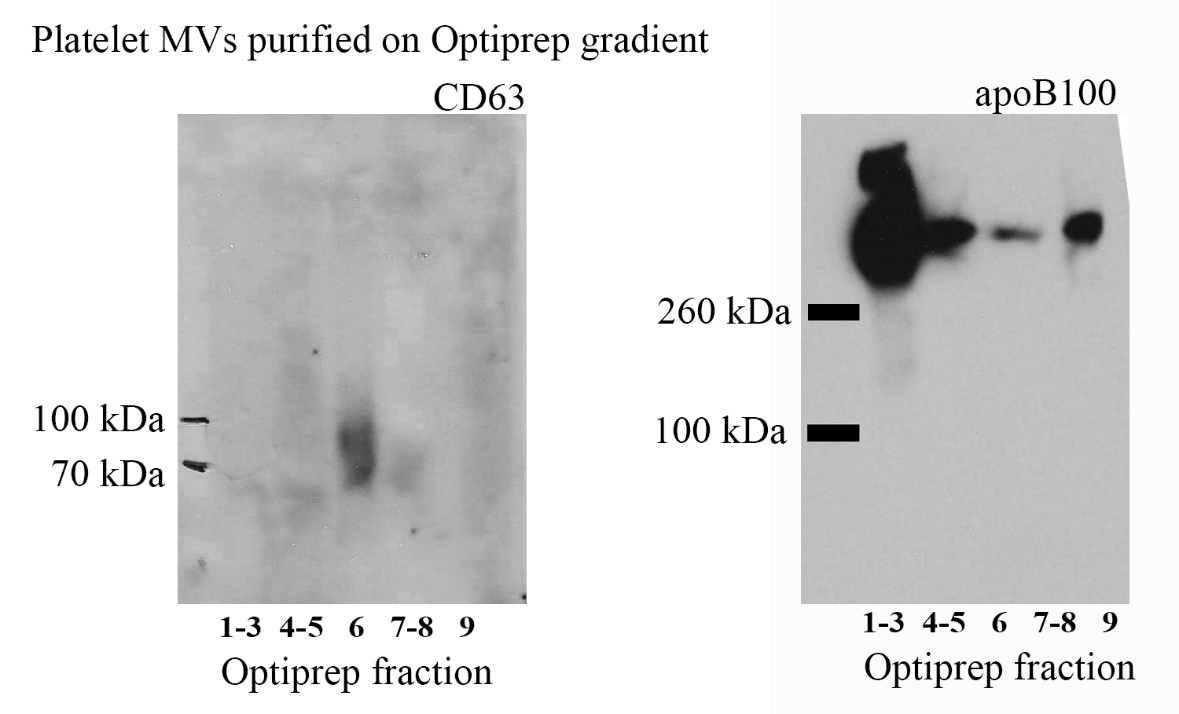


Supplementary Fig S12. Platelet concentrate-derived MVs purified on Optiprep^TM^ density-gradient. Western blots for CD63 and apoB. Uncropped gels.

*Supplementary Figure S13*


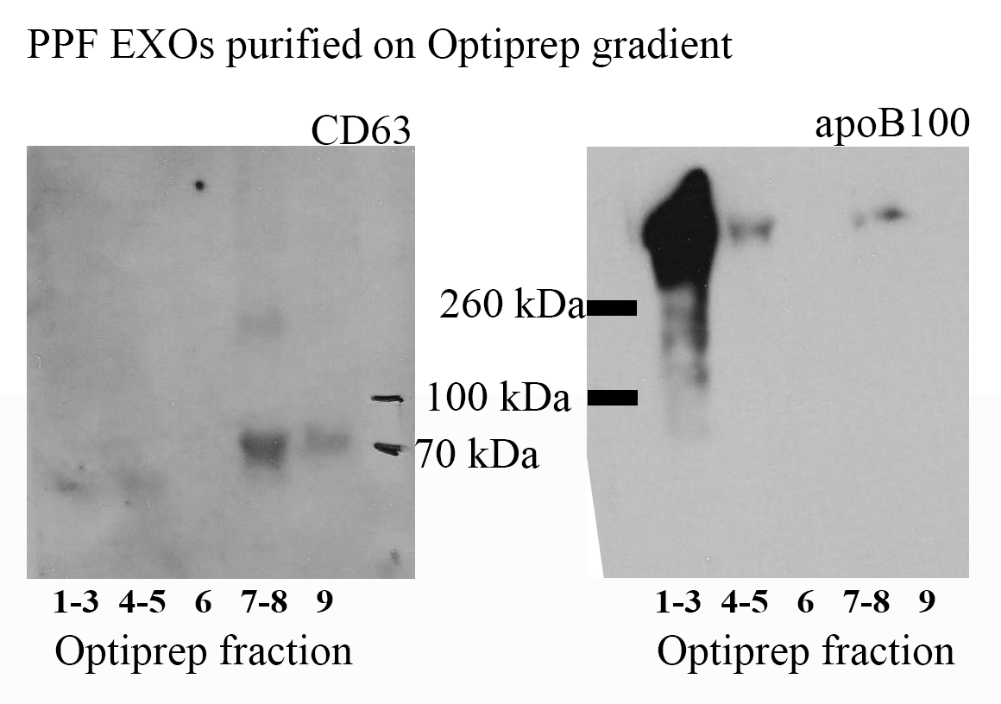


Supplementary Fig S13. Platelet-free plasma-derived EXOs purified on Optiprep^TM^ density-gradient. Western blots for CD63 and apoB. Uncropped gels.

*Supplementary Table S1*

|  | Fasting  (average ± SEM) | Postprandial  (average ± SEM) | p  (paired t-test) |
| --- | --- | --- | --- |
| Triglyceride (mM) | 1.24 ± 0.61 | 1.76 ± 0.75 | **0.014** |
| Total cholesterol (mM) | 4.28 ± 0.42 | 4.21 ± 0.39 | 0.270 |
| LDL-cholesterol (mM) | 2.08 ± 0.40 | 2.00 ± 0.37 | 0.093 |
| ApoB100 (g/L) | 0.74 ± 0.15 | 0.72 ± 0.13 | 0.048 |
| ApoA1 (g/L) | 1.50 ± 0.18 | 1.47 ± 0.17 | 0.282 |

Serum samples analyzed by routine laboratory measurement in fasting and postprandial state. Triglycerides increased in serum upon food intake, while apoB100 slightly decreased.

*Supplementary Table S2*

| Fasting MVs | | Postprandial MVs | |
| --- | --- | --- | --- |
| Acces | Protein | Acces | Protein |
| ALBU_HUMAN | Serum albumin | ALBU_HUMAN | Serum albumin |
| **APOB_HUMAN** | **Apolipoprotein B-100** | CO3_HUMAN | Complement C3 |
| CO3_HUMAN | Complement C3 | **APOB_HUMAN** | **Apolipoprotein B-100** |
| A2MG_HUMAN | Alpha-2-macroglobulin | A2MG_HUMAN | Alpha-2-macroglobulin |
| TRFE_HUMAN | Serotransferrin | TRFE_HUMAN | Serotransferrin |
| CO4B_HUMAN | Complement C4-B | CO4B_HUMAN | Complement C4-B |
| CO4A_HUMAN | Complement C4-A | CO4A_HUMAN | Complement C4-A |
| FIBB_HUMAN | Fibrinogen beta chain | FIBB_HUMAN | Fibrinogen beta chain |
| FINC_HUMAN | Fibronectin | APOE_HUMAN | Apolipoprotein E |
| IGHM_HUMAN | Ig mu chain C region | CERU_HUMAN | Ceruloplasmin |
| **APOA1_HUMAN** | Apolipoprotein A-I | APOA1_HUMAN | Apolipoprotein A-I |
| FIBA_HUMAN | Fibrinogen alpha chain | IGHM_HUMAN | Ig mu chain C region |
| CERU_HUMAN | Ceruloplasmin | FIBA_HUMAN | Fibrinogen alpha chain |
| CFAH_HUMAN | Complement factor H | FINC_HUMAN | Fibronectin |
| FIBG_HUMAN | Fibrinogen gamma chain | FIBG_HUMAN | Fibrinogen gamma chain |
| IGHG3_HUMAN | Ig gamma-3 chain C region | IGHG3_HUMAN | Ig gamma-3 chain C region |
| **APOE_HUMAN** | Apolipoprotein E | HPT_HUMAN | Haptoglobin |
| **APOA4_HUMAN** | Apolipoprotein A-IV | ACTB_HUMAN | Actin, cytoplasmic 1 |
| MUCB_HUMAN | Ig mu heavy chain disease protein | MUCB_HUMAN | Ig mu heavy chain disease protein |
| IGHG1_HUMAN | Ig gamma-1 chain C region | A1AT_HUMAN | Alpha-1-antitrypsin |

Proteins identified from fasting and postprandial MV samples by mass-spectrometry. The top 20 hits with the highest peptide coverage are listed. Apolipoprotein B has been identified using tandem MS in both pre and postprandial samples with 54 and 56 peptides, respectively, with sequence coverage of 18% and 19%. Protein identification is unequivocal. Based on peptide abundances apoB100 was among the 10 most abundant components of the samples.
